# Strengthening the future of science: Why joining a scientific society has never been more important

**DOI:** 10.1111/pbi.70145

**Published:** 2025-06-11

**Authors:** Peter R. Ryan, Liana G. Acevedo‐Siaca, Geraint Parry, Kailash Chander Bansal

**Affiliations:** ^1^ Research School of Biology The Australian National University Acton Australian Capital Territory Australia; ^2^ Horticulture and Product Physiology Wageningen University Wageningen Netherlands; ^3^ Association of Applied Biologists Wellesbourne UK; ^4^ Department of Biotechnology Guru Jambheshwar University of Science and Technology Hisar India; ^5^ Centre for Crop and Food Innovation Murdoch University Perth Western Australia Australia

Dear Editor,

Science is very much a collaborative process that builds upon previous findings. For this reason, scientists and engineers typically form associations and societies to meet and share information on topics of common interest. Thousands of local, national, and international societies exist to serve a wide range of disciplines at all academic levels. Indeed, some of these professional associations are centuries old (Schwartz *et al*., 2008).

Scientific societies benefit the professional lives of their members and nurture the next generation of researchers and professionals. They provide invaluable networking opportunities where students and early career researchers (ECRs) meet experienced researchers and receive encouragement and critical feedback in supportive environments. The confidence and performance of junior members is boosted by being part of a community (MIT Teaching and Learning Lab, 2023; Stachl and Baranger, 2020); excellence is recognized with prestigious awards, and senior members have opportunities to exchange research results and ideas and join the executive for leadership experience. Yet, scientific societies have experienced mixed fortunes over recent decades, with declining memberships and waning interest from students, ECRs, and senior scientists alike (Schwartz *et al*., 2008). Fewer members reduce the income and resources that fund activities, which compounds the problem further. The Society for Conservation Biology lamented the trends occurring in their organization in 2008:

Despite this maturation, scientific societies may now be poised on the precipice of oblivion. Many societies, failing to convince their constituents of the full value of their collective mission, are losing membership, and without members, a scientific society must either become a profit‐focused business or cease to exist. (Schwartz *et al*., 2008)

This decline has been a global trend across disciplines which suggests fundamental shifts in science and society are responsible. Indeed, the same trend appears to extend to non‐scientific, political, commercial and community associations (Khawaja, 2024; McKliney Advisors, 2024). Here, we offer reasons for the dwindling popularity of many biological societies and offer suggestions to reverse this trend. Finally, we highlight the valuable contributions scientific societies can make to the wider communities by providing trusted information in the many public debates that are currently shaping our future.

Why are established scientific societies finding it more difficult to attract and retain members? A range of issues undoubtedly influences whether any individual joins a professional society or not. However, we believe the declining fortunes of many biological societies can be linked to five over‐arching developments: the revolution in communication technologies, the momentous developments in genetics and molecular biology, the precarious employment opportunities for ECRs, the failure to adequately inform students and young scientists, and the recent COVID‐19 pandemic.

The headline events in any society's calendar are the annual meetings and periodic workshops and social gatherings. Traditionally, these events benefited members by facilitating the exchange of ideas and information and by providing access to the latest research. It is easy to forget that less than a generation ago the necessary task of keeping up‐to‐date with the scientific literature was laborious and time‐consuming. It required regular trips to the library to scan Chemical Abstracts™, Current Contents™, and the journals and books libraries could afford to stock. Research articles of interest that were not available in the local library needed to be obtained from other institutions or by requesting reprints from corresponding authors around the world by mail. Society meetings provided a welcome shortcut to this onerous process because information and research results were openly shared. The internet revolution lifted that burden. Very soon anyone could perform complex literature searches and access abstracts and research papers online from the comfort of their own offices. Suddenly, one of the primary incentives for joining a society, accessing the latest research, was less compelling.

During the same period, biology was experiencing a revolution of its own. To encompass the momentous developments in molecular biology and biotechnology, biological societies began to restructure and reorganize. Some changed their names, others combined with related groups, and many joined forces to convene joint annual conferences. These joint meetings continue today and are generally much grander affairs that provide quite different experiences from the old‐style meetings. The changes were not welcomed by everyone. Whereas society meetings traditionally occurred on university campuses with self‐catering and budget accommodation, the new‐style conferences require larger venues and professional organizers. The size and energy of these meetings are exciting for some but overwhelming for others. Anyone can attend these conferences, but society members benefit from lower registration costs and sponsored activities. Even so, the large society‐based conferences are expensive to attend and more competitive to secure speaking slots. Many people opt out of these society conferences and instead attend smaller workshops organized around specific research topics where there is no requirement or advantage in being a society member.

Another factor contributing to the declining fortunes of scientific societies has been the falling morale among early‐career STEM (science, technology, engineering and mathematics) professionals (Science and Technology Australia, 2021; Woolston, 2021). Job security preoccupies the minds of ECRs across disciplines. The shortage of permanent jobs in academia means that fixed‐term contracts are becoming the norm (OECD, 2021) The competition for continued funding is intense and often relies on research outcomes. But research is unpredictable. Bold hypotheses are regularly revised and unexpected hurdles can stymie the most exciting of projects and deflate the most promising of researchers. Indeed, precarious employment opportunities, modest starting salaries and the lack of stability to start families (Nature Editorial, 2023; Nordling, 2023) leave many ECRs disillusioned with their chosen careers and many abandon research altogether (Kang, 2023; Langin, 2022, 2024; Rothenberg, 2024). Stretched for time, money and energy, ECRs feel they must devote themselves to their research, which leaves little space for discretional activities such as professional societies.

Scientific societies tend not to promote themselves well. They rely too much on word‐of‐mouth to inform others about their activities. Each cohort of students moving through the system may be simply unaware of their existence and the advantages of joining up. Anecdotal observations drawn from the authors own four societies support this conclusion. First, the members of a society are not evenly spread among all the relevant universities and institutes. Instead, they are clumped so that disproportionally more members are linked with some institutes than others. Furthermore, we observe that if the head of a laboratory is a member of a society, then it is more likely that the students and ECRs in that laboratory will be society members as well. The opposite tends to be true as well: if the head of a laboratory is not a society member, then it is less likely that his/her students will be either. We conclude that some individuals and institutes nurture a culture that encourages participation in societies. Members of the other groups may simply be unaware of what societies are and what they have to offer.

More recently, the COVID‐19 pandemic restricted meetings and travel and forced us all to work online. Membership subscriptions plummeted because the case for joining a society that could not meet face‐to‐face became less persuasive. For instance, the Australian Society of Plant Scientists (ASPS) experienced a 27% fall in membership over consecutive years from 2018/19 to 2019/20. Similar decreases were reported by the American Society of Plant Biologists (ASPB) where the average number of professional members between 2021 and 2024 fell by 32% compared with the previous 5‐year average. Not surprisingly, the novelty of Zoom™ meetings and online conferences soon wore thin so that when COVID‐19 restrictions gradually lifted, the benefits of being a member of an active local society became obvious. Face‐to‐face events were enthusiastically embraced when they were available. As a result, the post‐COVID‐19 period triggered a resurgence in membership in some societies, including the Scandinavian Plant Physiology Society, the Portuguese Society of Plant Biology and the ASPS which saw a 91% bounce in members between 2020 and 2022. Most eager of all to join up were anxious graduate students, no doubt keen to get their research projects back on track. The communication technologies that developed so rapidly during the COVID‐19 period are here to stay and remain useful options for meetings in the future.

How, then, can the fortunes of scientific societies be improved? The key is nicely distilled by Schwartz *et al*. (2008):

To define scientific societies solely as the vehicle for producing a journal, or an annual meeting, is to invite valuation of a society on narrow economic terms. People can usually access the same goods and minimize expense through other avenues; thus, not being a member is the cost‐minimizing choice. For scientific societies to thrive in the 21st century, they must mean more to members than simply the source of a journal or a meeting. (Schwartz *et al*., 2008)

First and foremost, financial security is essential for any organization to flourish. Funds are required to resource events, maintain websites, promote participation in meetings and workshops, accommodate invited speakers, subsidize student travel, and provide meaningful awards. Most societies survive on annual membership fees, which they work hard to keep low and not prohibitive. Student payments are minimal and often fully recouped by attending a single meeting due to travel grants and subsidies. Annual fees are sometimes supplemented by commercial subscriptions, private donations, and modest profits generated by functions. A small number of societies publish scientific journals, which can be very profitable and a welcome source of kudos (e.g. American Society of Plant Biologists, Society for Experimental Biology, Association of Applied Biologists, Japanese Society of Plant Physiology, British Society for Plant Pathology, Scandinavian Plant Physiology Society, and Brazilian Society of Plant Physiology). Some even offer reduced publication costs to society members linked with them, which is an added advantage. However, the business case for academic publishing has changed over the past 30 years (Ghasemi *et al*., 2022). Numerous academic journals began appearing once publishers realized the open‐access model avoided many of the costs associated with traditional publishing: the articles are free, the peer reviews are free, and production costs for online publications are low. Additional competition came from the plethora of predatory or vanity publishers that emerged to exploit the ‘pay‐to‐publish’ model with scant regard to quality (Scicluna, 2024). Launching a new journal is not an option for most societies, and nor is it essential for a society to be successful. Annual membership fees are sufficient as long as the membership is large enough. The key challenge, therefore, is to boost and maintain subscriptions. We now discuss how this might be achieved by highlighting the roles of advertising, lobbying, and offering activities that are seen as desirable and beneficial.

The first of these ideas to recruit members is simple: societies need to advertise themselves in all suitable institutions regularly so all prospective members are informed of what a society is and what they do. They should be aware of research showing that members of societies tend to perform better, suffer fewer mental health issues, and remain in active research for longer than those not society members (MIT Teaching and Learning Lab, 2023; Stachl and Baranger, 2020). This information will attract new students and assuage the concerns of ECRs who are increasingly anxious about spending time away from the bench. Indeed, the benefits of membership are so apparent and clear that tactful lobbying by societies might persuade universities and research institutes to subsidize student participation in a society and even convince funders to include membership fees in their research grants.

Societies should engage with their members more regularly through newsletters and offering different types of events. We discussed above how large annual conferences in convention halls with parallel sessions are overwhelming and unsatisfactory for members who prefer smaller meetings that focus on specific research topics. Some societies were sensitive to this feedback and responded by scheduling different styles of gatherings. One example is the Federation of Canadian Plant Science Societies, which is an umbrella organization representing seven Canadian societies linked with plant science and agriculture. In three out of four years, those societies hold their own annual meetings separately but, in the fourth year, they combine for a large conference named ‘Plant Canada’ (https://www.plantcanada.ca/index.html). The Australian society, ASPS, recently began trialling a three‐year meeting cycle for the same reason. For two years, ASPS holds its own meeting and on the third, it joins with related societies from Australia and New Zealand to convene a much larger combined conference called ComBIO. Even within societies, smaller workshops could be organized throughout the year to meet certain needs. Separate social events without a formal scientific program can engender a sense of community away from the pressures of work. Most of us will agree that it is often in these more relaxed gatherings that people find common interests and exchange ideas more freely.

Scientific societies need to respond to the changing needs of their members. Securing a job is an anxiety experienced by most young scientists. A greater emphasis on career development and employment opportunities is bound to be a welcome initiative. Science societies naturally focus on research and academia. Yet, only a minority of science graduates become professional researchers and academics. Most join industries, businesses, or become public servants and teachers. While some scientific societies promote the importance of education and outreach, there is a compelling case for them to cater for a wider diversity of career choices. Links with local companies and industries could be fostered with meet‐and‐greet sessions, information seminars, and tours of local employers. These could be combined with the annual meetings or held as separate networking events. These initiatives are likely to be viewed very favourably by students and ECRs. They may also generate closer ties with industries and businesses whereby the societies offer annual seminars on the latest developments in plant science and biotechnology in exchange for corporate sponsorship. These initiatives, together with those ideas discussed above, provide ways that societies can attract new members and improve retention.

We emphasize that scientific societies have a responsibility to our communities in addition to their members. The world is confronted with an unprecedented list of ecological and social challenges. Climate change, resource depletion, pandemics, social unrest, and artificial intelligence are already impacting our lives. For the global community to manage the changes and meet the challenges that arise requires our leaders to make decisions based on the most reliable information and modelling. Despite this, science and scientists have been experiencing a crisis of trust and credibility in segments of the population (Mill and St. Clair, 2025; UNESCO, 2022). It should be a concern to us all that in parliaments, legislatures, and town halls around the globe, scientific advice is ignored, derided, or worse, replaced with half‐truths and falsehoods. The bizarre conspiracy theories and home remedies that circulated during the COVID‐19 crisis (Hartman *et al*., 2021; Lynas, 2020) would have been laughable were it not for the harm they could cause to a confused and nervous population. Similarly, the misinformation concerning genetically engineered crops has stymied efforts to improve global food security and overcome disease and micronutrient deficiencies (Klümper and Qaim, 2014; Smyth, 2020). Even now, consensus opinions issued by the Intergovernmental Panel on Climate Change (IPCC) are questioned or ignored by some leaders (Hudson, 2016; McCarthy, 2014; Yandell and Jaramillo, 2004). There needs to be pushback. The deliberate spread of misinformation and ‘alternative facts’ to bolster personal, political, or commercial interests has to be systematically contested in open debate. We scientists cannot continue to allow society to descend into the nightmarish scenario of being unable to distinguish science from pseudoscience or facts from opinions pedalled by vested interests. A culture of critical thinking needs to be fostered, and scientific societies are part of the answer for achieving this.

We argue that scientific societies should engage more directly in important public debates by providing trusted, evidence‐based information. Their conclusions are based on the best science that can be tested and updated as new information becomes available. The same standards should be demanded of anyone of influence who proclaims outlandish and controversial views in the public arena.

As well‐informed individuals, any of us can add our voices to the information storm, but the impact will be minimal if no one hears us in the maelstrom. Scientific societies that calmly and firmly express the collective opinions of experts can more persuasively inform the public and policymakers on specific matters. Many societies already engage this way, but much more is needed. For instance, the French Academy of Agriculture informs the government and the public on all aspects of agricultural sciences (https://www.academie‐agriculture.fr/publications/articles). The American Society of Plant Biologists (ASPB) comments publicly on government policies that affect the wider science community (https://blog.aspb.org/aspb‐reaffirms‐its‐commitment‐to‐diversity‐and‐inclusion/) and has a dedicated Science Policy Committee that regularly addresses the US Congress and Executive Branch on matters affecting science and agriculture. More powerful still are collectives that advocate on behalf of many societies in an objective and apolitical manner. Examples include the ASPB, which recently joined with 40 other scientific societies to lobby Congress on the links between research and food security; the European Federation of Academies of Sciences and Humanities, which represented multiple organizations to support students and researchers fleeing the conflict in Ukraine (https://allea.org/support‐for‐ukraine/); the European Plant Science Organization representing 70 member organizations from 31 countries in presentations to the European Commission and national politicians on local and global science policy; and the Global Plant Council (https://globalplantcouncil.org) which contributes to public forums and policy development on behalf of 24 plant science societies. Similarly, Science and Technology Australia is a peak body representing more than 140 organizations that contribute to public debates and science policy development. These efforts are important, but engagement by societies this way must increase and be sustained.

There have always been compelling reasons for joining a scientific society. Yet, societies need to innovate so that the benefits they deliver continue to attract members from each new generation of scientists as their needs and expectations change. It is essential that these organizations grow and remain active because their engagement in public debates has never been more important.
